# Molecular mechanisms of uterine incision healing and scar formation

**DOI:** 10.1186/s40001-023-01485-w

**Published:** 2023-11-08

**Authors:** Qing Sun, Le Tang, Dan Zhang

**Affiliations:** 1 Shenyang Women’s and Children’s Hospital, Shenyang, 110000 China; 2 Obstetric Department, Shenyang Women’s and Children’s Hospital, Shenyang, 110000 China

**Keywords:** Uterus, Caesarean delivery, Wound healing, Scar, Molecular mechanism

## Abstract

Wound healing is a tandem process involving inflammation, proliferation, and remodeling, through which damage is repaired and ultimately scar tissue is formed. This process mainly relies on the complex and extensive interaction of growth factors and cytokines, which coordinate the synthesis of various cell types. The loss of normal regulation in any part of this process can lead to excessive scarring or unhealed wounds. Recent studies have shown that it is possible to improve wound healing and even achieve scar-free wound healing through proper regulation of cytokines and molecules in this process. In recent years, many studies have focused on accelerating wound healing and reducing scar size by regulating the molecular mechanisms related to wound healing and scar formation. We summarized the role of these factors in wound healing and scar formation, to provide a new idea for clinical scar-free healing treatment of uterine incisions.

## Introduction

Whenever the integrity of an organ is compromised, multiple essential functions of the organ are threatened. Wounds, which usually result from tissue damage, heal through a complex series of processes involving the interaction between growth factors and cytokines [1], and the ultimate goal is to replace damaged cells and restore the integrity of the organ. The mechanism of wound healing can be divided into three successive but overlapping phases; inflammation, proliferation, and tissue remodeling [2]. The outcome of wound healing in adults is the formation of scar tissue, which is a normal physiological reaction and part of the natural healing process after injury. The formation of scar tissue involves the interaction of cells, growth factors, cytokines, and the extracellular matrix (ECM).

Poor healing of uterine scars after caesarean delivery is a common complication that seriously threatens maternal health. Unfortunately, there is no good treatment and prevention method for the poor healing of uterine incision scars, and up to 30% of women have ultrasound evidence of muscular disruption at the site of a previous caesarean delivery 6–12 months after surgery, and lower uterine thickness < 2.5 mm was associated with a higher risk of uterine rupture during delivery [3]. At present, researchers can reduce the incidence of poor uterine incision healing through the selection of uterine incision, improving suture methods, preventing uterine incision infection, selecting suture materials, and other aspects, but there have been relatively few studies on the histopathology of uterine incision healing after caesarean delivery and the molecular signalling pathways affecting healing.

## The definition of wound healing

Wound healing is a conserved and complex multistep process involving many blood and parenchymal cells, soluble mediators, and ECM proteins. The complexity of these steps makes it susceptible to perturbations at many levels, and anything that can affect physiological responses and cell function can negatively affect wound healing, leading to poor tissue repair or excessive scarring, which can result in pathological conditions that impair organ function. In 1971, Burrington first proposed the concept of "scar-free healing" [4]. Transforming growth factor-β(TGF-β), as a therapeutic target or therapeutic means to promote wound healing and/or reduce scar formation, has achieved great results in experimental and clinical trials [5–8]. In addition, the connective tissue growth factor is, the most important molecular factor involved in scar formation. Basic fibroblast growth factor (bFGF), platelet-derived growth factor (PDGF), and vascular endothelial growth factor (VEGF) also participate in this process [9].

## Uterine incision scar formation during caesarean delivery

Caesarean delivery can cause uterine damage, resulting in excessive fibroblast activation and the continuous secretion of collagen, which ultimately leads to the formation of a uterine scar. Excessive collagen deposition hinders the proliferation, differentiation, and migration of the original cells of the uterus, and causes collagen scarring in the endometrium [10, 11]. Excessive wound healing is often due to the dysregulation of signals during remodeling, which leads to scar formation. However, this may be caused by the overexpression or reduction of essential growth factors and cytokines during wound healing [12]. The increased ratio of TGFβ1/β3 can reduce scarring and fibrosis, and altered expression of connective tissue growth factor (CTGF) may be a factor in abnormal scar formation and uterine rupture in the lower uterine segment after caesarean delivery. bFGF deficiency is associated with a decrease in collagen deposition in the wound site and thicker scabs. Changes in the expression of tumour necrosis factor-α (TNF-α), VEGF, and PDGF in human muscle layer smooth muscle cells during uterine rupture are related to the healing process in the uterus [13]. Therefore, final scar formation and collagen deposition during uterine wound healing may be related to changes in growth factor expression in the uterine incision. The environment and intrinsic characteristics of foetal and adult wounds were compared, and compared with those in adult scar healing, the levels of interleukin-6 (IL-6) and, interleukin-10 (IL-10) and epidermal growth factor (EGF) in foetal scar healing were decreased, while the levels of IL-10 and EGF were increased. TGF-β3 expression was significantly increased, and TGF-β1 expression was decreased, which further elucidates the mechanism related to scar-free healing in foetal wounds [14]. Although it remains to be further demonstrated whether adult uterine incisions have the same molecular mechanism, it is clear that the process of good scar healing and tissue function recovery is controlled by growth factors and cytokines.

## Molecular factors associated with wound healing

### TGF-β family

TGF-β mainly signals through the Smad pathway, and regulates inflammation, morphogenesis, angiogenesis, collagen generation, and cell differentiation. The TGF-β/Smad pathway is closely related to scarring and fibrosis, and its activation can induce excessive fibroblast proliferation and excessive ECM deposition. This pathway is initiated by the phosphorylation of Smad2 and Smad3 via the TGF-β receptor protein, which forms complexes with Smad4 and translocates to the nucleus. This translocated heteropolymer complex can regulate profibrotic genes and promote the differentiation of fibroblasts into myofibroblasts [15]. This complex is dephosphorylated by phosphatase, causing Smad4 to move back into the cytoplasm, where it forms another complex. If the TGF-β receptor is still active, the attenuation of this pathway can occur through the activation of Smad6 and Smad7, which bind to the TGF-β receptor and dephosphorylate Smad2 and Smad3. In addition, Smad7 can also introduce ubiquitin ligase into the receptor complex. TGF-β1 induces the expression of Smad7, which functions as a negative feedback loop. Overexpression of Smad7 causes cells to lose their responsiveness to TGF-β1, which has anti-inflammatory effects even in inflammatory diseases. TGF-β is a multifunctional growth factor, because the expression of different isomers has different effects on the wound. During wound healing, TGF-β promotes the formation of connective tissue, and regulates inflammation, proliferation, and re-epithelialization, Its three isomers, TGF-β1, TGF-β2, and TGF-β3, play important roles in wound contraction and scar formation. TGF-β1 is the most prevalent and biologically relevant isomer and is associated with excessive scarring and fibrosis in wounds. Although TGF-β3 is homologous to TGF-β1, it has an antifibrotic effect on skin tissue. In the early stage of wound healing, the concentration of TGF-β3 is higher than that of TGF-β1, and the concentration of TGF-β1 increases after epithelization [5]. TGF-β is a chemokine produced by endothelial cells that promotes the production of other growth factors, such as EGF and insulin-like growth factor (IGF). In addition, the EGF and TGF-β1 pathways can stimulate each other, and their interaction can inhibit the proliferation of endothelial cells. When bound to specific ligands on cell surface receptors, TGF-β promotes the production of keratinocytes, fibroblasts, neutrophils, and macrophages, and then triggers the release of proinflammatory cytokines such as IL-1α and β, IL-6, and TNF-α. Second, TGF-β can directly induce the expression of α smooth muscle actin (α-SMA) in fibroblasts, thus promoting their differentiation into myoblasts. TGF-β2 has similar activity to TGF-β1, and its downregulation can reduce scar formation [5]. Wound treatment with antibodies against TGF-β1 and TGF-β2 has been shown to restore the skin structure to a state similar to that of uninjured skin [16], suggesting that inhibiting or downregulating TGF-β1 and TGF-β2 may reduce the occurrence of scarring. In addition, TGF-β1 expression levels were different between youths and adults, and the expression levels in adults were higher. Penn's research on the role of TGF-β1 in wound healing suggests that changes in TGF-β1 are related to the severity of scarring, and TGF-β1 upregulates collagen genes and genes that promote collagen expression, such as membrane proteins [17]. In summary, TGF-β activity may contribute to wound contraction and closure, and is also associated with severe scarring and increased fibrosis. In recent years, more and more studies have focused on the uterus. Yao et al. [18] found that BMSC-derived Exo may promote endometrial repair through the TGF-β1/Smad signalling pathway. There is also evidence to support that TGF-β1 is strongly associated with the onset and progression of intrauterine adhesion (IUA) and is considered an early risk factor for disease recurrence [19]. In addition, it has been found that TGF increases the proliferative capacity of endometrial and muscle cells, promotes the regeneration of the microvascular system, and restores the ability of the endometrium to receive embryos and support their development to a viable stage, through the transplantation of collagen/BM-MSCs constructs [20]. Further study of TGF-β, its downstream signals and the role of crosstalk signals may provide new therapeutic targets for reducing scarring and fibrosis.

### CTGF

CTGF is a 38-kDa protein with a four-nodule structure that is the second member of the CCN family and, also known as CCN2. Previous studies have shown that CTGF promotes the adhesion of human vascular endothelial cells, lung epithelial cells, and fibroblasts. CTGF adhesion is mediated by the phosphorylation of focal adhesion kinase by binding to fibrinoconnexin, cell surface proteoglycans, integrins, and extracellular regulated protein kinase, and ultimately enhances focal adhesion by activating F-actin, Paxillin, and RhoA. CTGF can activate fibroblasts, induce fibroblasts to differentiate into myofibroblasts and stimulate collagen deposition and ECM protein remodelling, leading to tissue fibrosis and remodelling. CTGF can also induce the expression of cytokines, such as TGF-β and VEGF, and increase CTGF expression [21]. Thus, the expression of CTGF involves several positive feedback loops. CTGF is involved in cell proliferation, migration, and differentiation, and can directly promote the progression of fibrosis, or indirectly participate in fibrosis as a downstream factor of TGF-β. CTGF is associated with tissue repair and almost all fibrosis pathology, such as scleroderma, atherosclerosis, pulmonary fibrosis, renal fibrosis, and benign interstitial tissue growth. Studies suggest that CTGF can synergically induce persistent fibrosis in the presence of TGF-β, and exacerbate ECM production under other fibrosis induction conditions. TGF-β1 mediated overexpression of CTGF has been confirmed in airway smooth muscle cells [22]. The application of CTGF or TGF-β alone to the subcutaneous tissue of mice resulted in the transient formation of granulation tissue, while the administration of both CTGF and TGF-β resulted in the long-term formation of granulation tissue [23]. CTGF has been localized in many different tissues, including the normal uterus [24] and leiomyoma [25]. Pollio confirmed that CTGF may be the cause of abnormal scar formation in the lower uterine segment after caesarean delivery, as well as the cause of uterine rupture through changes in granulation tissue development and angiogenesis [26]. Therefore, we can improve the development of granulation tissue and promote angiogenesis by regulating CTGF, thus reducing the degree of scarring in the lower uterine segment.

### bFGF

FGFs are a 23-member fibroblast growth factor family that are released by a variety of cell types and interact with a set of receptors that exhibit tyrosine kinase activity. The most typical FGF in wound healing is FGF‐2 or bFGF. bFGF is a powerful mitogen that promotes the proliferation of fibrocytes, vascular endothelial cells, and smooth muscle cells. BFGF is expressed by many cells involved in wound healing, such as macrophages, endothelial cells, and muscle smooth muscle cells, and plays an important role in tissue regeneration and wound healing. bFGF upregulates the expression of multiple cytokines through the PKC/PI3K-AKT/MAPK signalling pathway and is present in the basement membrane and endovascular subcutaneous ECM in normal tissues. Ortega concluded that wound healing was delayed in bFGF knockout mice [27]. During scar-free foetal wound healing, the overall expression of bFGF is downregulated [28]. It is hypothesized that heparin sulfate degrades and activates bFGF during wound healing and tumour development in normal tissues, thereby mediating the formation of new blood vessels. In animal models, bFGF deficiency has been shown to lead to a reduction in collagen deposition and thickening of scabs at the wound site. The use of bFGF is certified for safe use and has been used in the treatment of ulcers and burns. bFGF has also made significant contributions to the fields of nerve, muscle, and bone regeneration [29], showing its outstanding regenerative potential. Previous studies have shown that bFGF is essential for endometrial cell proliferation, remodelling and angiogenesis during the menstrual cycle [30]. However, the level of endogenous bFGF induced during wound healing is too low to promote angiogenesis and wound repair, which highlights the idea of exogenous administration of bFGF. However, the disadvantages of exogenous administration include high diffusion, a short half-life, and side effects, which require repeated doses of recombinant bFGF in vivo. Studies have shown that collagen membranes loaded with recombinant bFGF containing the collagen-binding domain (CBD) to construct collagen-targeted bFGF delivery system can improve the regeneration of the endometrium, muscle cells, and blood vessels in model rats [31]. A recent study constructed an umbilical cord mesenchymal stem cells (UC-MSCs) with overexpression of basic fibroblast growth factor (UCMSC-bFGF), which was found to be able to accelerate the regeneration of the damaged uterine wall by inhibiting the acute inflammation in the early stage after uterine injury, and accelerating the regeneration of the damaged uterine wall, and repairing the whole layer of defective uterine wall in a rat model [32].

### PDGF

PDGF is a major mitogenic agent, which is part of family of homologous or heterodimeric growth factors with, i.e., four different polypeptide chains (PDGF-A, -B, -C, -D) that can form disulfide to create five homodimeric or heterodimeric glycoproteins (PDGF-AA, -BB, -CC, -DD, -AB). PDGF is a growth factor that has a chemotactic effect on cells entering healing skin wounds. This factor also promotes ECM production and fibroblast proliferation. PDGF stimulates fibroblasts to contract the collagen matrix and induces a myofibroblast phenotype in these cells [33]. PDGF can promote wound healing by stimulating mitosis and chemotaxis in fibroblasts and smooth muscle cells, chemotaxis in neutrophils and macrophages, and the generation of various matrix molecules, such as fibrin, collagen, proteoglycan, and hyaluronic acid [34]. PDGF is an important angiogenic factor, which can induce angiogenesis in various tissues. On the one hand, PDGF promotes the growth and proliferation of vascular endothelial cells. On the other hand, vascular outer membrane cells and vascular smooth muscle cells are induced to transform into new blood vessels to regulate the growth and stabilize the formation of blood vessels. In the myocardial infarction model, PDGF-BB can preferentially stimulate the growth of microarteries [35], and in a mouse corneal angiogenesis model and a rabbit hind limb ischemia model, PDGF-BB synergizes with bFGF to promote angiogenesis [36]. PDGF may play an important role in the occurrence and development of early endometrial cancer by inducing angiogenesis [37]. In a mouse endometrial injury model, PDGF treatment promoted endometrial epithelial proliferation, migration, and tube formation, and facilitated re-epithelialization and endometrial repair, also suggesting again that PDGF may be a candidate for endometrial injury repair [38].

### VEGF

The VEGF family currently includes VEGF-A, VEGF-B, VEGF-C, VEGF-D, VEGF-E, and placental growth factor (PLGF). These factors exerts their biological effects by binding to three different transmembrane tyrosine kinase receptors; VEGFR-1, VEGFR-2, and VEGFR-3. The VEGF growth factor family is expressed in many cell types in the wound bed, including endothelial cells, fibroblasts, and smooth muscle cells. Tissue injury leads to the destruction of capillaries and hypoxia, resulting in increased expression of hypoxia-inducible factor 1-α (HIF-1α). Subsequently, the expression of VEGF and its receptors at the wound site is upregulated to stimulate angiogenesis [39]. VEGF is the most important angiogenic factor in tissue wound healing. This factor is not only necessary in the early stage of angiogenesis but also plays a role in angiogenesis. VEGF expression is downregulated in non-scar foetal trauma, and the application of exogenous VEGF leads to scar formation [40]. VEGF is crucial for endometrial angiogenesis and re-epithelialization. Wang et al. reported that the proliferation of Huatong mesenchymal stem cells into endometrial and myometrial cells may be caused by the production of IGF-1 and VEGF [41]. The injection of collagen combined with VEGF into sites around the uterine scar in a rat model could promote the remodelling of the scarred uterus, including the regeneration and vascularization of the endometrium, and improve pregnancy outcomes [42].

### IL-6

IL-6 is a multipotent cytokine in the immune system that plays an important role in wound healing. IL-6 is involved in regulating the fibrotic network composed of fibroblasts, macrophages, keratinocytes, and vascular endothelial cells, which is closely related to the formation of hypertrophic scars. High levels of IL-6 can change the biological behaviour of wound healing cells, disrupt the synthesis-degradation balance of ECM, and promote hypertrophic scar formation. IL-6 can affect scar formation by regulating the biological behaviour of cells. The classical signalling pathway mainly mediates the anti-inflammatory activity of IL-6, while the trans-signalling pathway shows proinflammatory activity and is related to various pathological changes in the body. Fibrosis diseases in various organs such as the liver, kidney, and lungs are closely related to the IL-6 trans-signalling pathway. The occurrence of fibrosis in these organs can be significantly reduced by blocking the IL-6/STAT3 signalling pathway [43, 44]. In vitro studies have shown that CD206^+^ macrophages have antifibrotic effects on fibroblasts by participating in the parasecretory mechanism of IL-6 [45]. IL-6 is involved in postpartum wound healing by stimulating the migration and proliferation of keratinocytes and mediating PDGF to stimulate fibroblast proliferation [46]. Liechty [37] observed that when adult and foetal free skin grafts were applied to full-thickness skin defect wounds on immunodeficient mice, the foetal skin grafts expressed IL-6 at 4 h after surgery, and no IL-6 was detected at 12–72 h after surgery, while the adult skin grafts continued to highly express IL-6 at 0–72 h after surgery. The injection of exogenous IL-6 recombinant protein into the full-layer skin defect in mice with foetal skin grafts resulted in scar formation. A decrease in inflammatory cytokines such as IL-6 may inhibit inflammation during foetal wound healing. Reducing inflammation may provide a beneficial environment for scar-free wound healing.

### IL-10

IL-10 is a multifunctional cytokine of multicellular origin that regulates cell growth and differentiation and is involved in inflammation and immune responses. IL-10 is one of many inflammatory and immunosuppressive factors. IL-10 activity is thought to be mediated by the STAT3-mediated (IL-10R/STAT3) signalling pathway, which is mediated by its receptor (IL-10R). It was first discovered that this factor is synthesized and secreted by mouse CD4^+^Th2 cells, and can inhibit the synthesis of IFN-γ by Th1 cells, thus preventing the proliferation of antigen-specific T cells, inhibiting presentation by antigen-presenting cells, and inhibiting the synthesis and expression of inflammatory cytokines and inflammatory mediators. IL-10 mitigates the harmful effects of lipopolysaccharide-induced wound healing by modulating the TLR4/NF-κB pathway in dermal fibroblasts, reducing scar contracture, scar formation, and skin fibrosis. IL-10 is a candidate for scar improvement therapy based on preclinical studies. By comparing the skin and soft tissue defects of IL-10 knockout mice and wild-type mice, it was found that IL-10 could promote wound healing and reduce scar formation by inhibiting the inflammatory response [47]. King [48] found that IL-10 could prevent scar formation by inhibiting the synthesis of IL-6, IL-8, and TGF-β. Other experiments have shown that IL-10 overexpression reduces the inflammatory response to injury, creating an environment that is conducive to regenerative adult wound healing [49]. In summary, IL-10 is a potential agent to prevent hypertrophic scarring by regulating fibroblast proliferation, migration, and ECM synthesis. Inhibiting inflammation is also the main reason why IL-10 is used to prevent scar formation. However, its short half-life and poor targeting result in the poor efficacy of IL-10 in preventing and treating scar formation. However, some experimental results show that local injection of IL-10-modified adipose mesenchymal stem cells (IL-10-ADMSCs) can maintain a high level of IL-10 in the wound for 3–5 days, which solves the problem of the short half-life of IL-10 in vivo. The effect of IL-10-ADMSCs on inhibiting wound inflammation and preventing hypertrophic scar formation has also shown that this is feasible [50]. Elevated levels of IL-10 have been identified as a key factor in the development of endometriosis, and localized secretion of IL-10 by plasmacytoid dendritic cells promotes the development of endometriosis through pathologic angiogenesis in the early stages of the disease [51]. It is reasonable to believe that uterine incision healing and scar formation can be improved by regulating IL-10.

## Discussion

Uterine injury, the most common postoperative complication in obstetrics and gynecology, and the complete recovery of the endometrial and myometrial tissues inevitably affects maternal health and uterine fertility. For example, IUA, for which hysteroscopy, hormonal therapy and the use of intrauterine devices have been attempts to address over the past few decades, have all shown some shortcomings. Then again, the global prevalence of serious obstetric complications such as scarred pregnancies, placental implantation, and uterine rupture has been increasing rapidly and is even more life-threatening for the mother. Significant progress has been made in understanding the wound healing process over the past few years, and our understanding of signalling during wound healing has increased; a large number of growth factors and cytokines are present at the wound site, and the dynamics of their expression exhibit characteristic spatial and temporal regulation, and altered patterns of growth factor expression have been associated with impaired wound healing, which has guided the development of a wide range of therapeutic approaches that accelerate wound closure and contraction. Although the use of growth factors in the treatment of wounds has shown positive results in restoring skin structure and the function of subepidermal components of the skin, the mechanism of wound healing in the muscular layer, which determines the future morphologic and functional behaviour of the scarred uterus, remains a major challenge, but there is still no effective anti-scar treatment. On the one hand, this may be due to the complex interactions between multiple cell types, growth factors and cytokines, and gaps in our understanding of the dynamics of these molecules. Most importantly, changes in the levels of one factor may affect other growth factors and cytokines. It is well known that wound repair is a complex, tightly controlled process that requires the coordinated activity of soluble factors and cells involved in inflammation, tissue deposition, and tissue remodeling, and that alterations in each of these steps may lead to a different or impaired healing process. The involvement of TGF-β, CTGF, bFGF, PDGF, VEGF, IL-6, and IL-10 in the etiology of wound healing is particularly emphasized. On the other hand, abnormalities in the expression of growth factor genes involved in the process of uterine scar formation may represent patient-specific features that increase the risk of complications associated with keloid uterus. Clinical applications using growth factors to promote wound healing are currently being investigated, and with the development of recombinant growth factors, the short lifespan of these molecules is no longer a major challenge, but there is still a need for better delivery systems that are able to detect microenvironmental conditions in the wound and respond to temporal and concentration precision by releasing growth factors. Despite advances in the understanding of the science of wound healing, many more steps remain to be discovered and elucidated.

## Conclusion

Uterine incision healing is a complex process involving multiple stages, including inflammation, cell proliferation, ECM remodeling, and angiogenesis. In recent years, significant progress has been made in understanding the molecular mechanisms of uterine incision healing, particularly the roles of growth factors and cytokines. The interplay between these growth factors and cytokines collectively regulates the various stages of uterine incision healing. However, there are still unknown areas in the molecular mechanisms of uterine incision healing that require further investigation. Furthermore, researchers need to delve deeper into the roles of these molecules in different types of uterine incision healing. Overall, studying the molecular mechanisms of uterine incision healing contributes to a better understanding of the process and provides a theoretical basis for the development of novel therapeutic approaches.

## Data Availability

The datasets generated during and/or analysed during the current study are available in the Pubmed repository, [PubMed (nih.gov)]; the Huiyimd repository, [(huiyimd.com)]; the Wanfangdata repository, [(wanfangdata.com.cn)] et al.
